# Solution and precipitation based radical polymerization of renewable vinyl lactones in renewable solvents†

**DOI:** 10.1039/d5ra02151k

**Published:** 2025-06-25

**Authors:** Dimitrios Apostolidis, William E. Dyer, Clemens A. Dransfeld, Baris Kumru

**Affiliations:** a Aerospace Structures & Materials Department, Faculty of Aerospace Engineering, Delft University of Technology 2629 HS Delft Netherlands b.kumru@tudelft.nl

## Abstract

Sustainable polymers are essential to reducing the environmental impact of conventional plastics. While the use of renewable feedstocks plays a significant role, the adoption of green processes, including sustainable solvent selection and efficient polymer purification, is equally essential. This study presents a green synthesis route for polymers based on two renewable vinyl lactone monomers: α-methylene-γ-valerolactone (MeGVL) and α-methylene-γ-butyrolactone (MeGBL). Polymerization was performed in renewable solvents as Cyrene®, γ-valerolactone, and 2-methyltetrahydrofuran *via* solution and in biobased alcohols through precipitation methods. While solution polymerization requires additional purification step through polymer precipitation, precipitation polymerization enables efficient polymer recovery and solvent reuse. The resulting polymers made *via* precipitation polymerization exhibit properties with glass transition temperatures of 99 °C (polyMeGVL) and 94 °C (polyMeGBL), and visible light transmittance over 96% between 450–700 nm of both polymer films of thickness around 100 μm. Water contact angles of the films were 62° for polyMeGVL and 51° for polyMeGBL showing difference despite having a similar chemical composition. These results highlight a scalable, low-impact pathway for producing commodity polymers entirely from renewable resources.

## Abstract 2

Acrylic-type renewable vinyl lactone polymers are made *via* radical polymerization in bio-based solvents. A precipitation route provides high-purity materials with high optical transparency, highlighting a scalable path for green polymer production.

## Graphical abstract

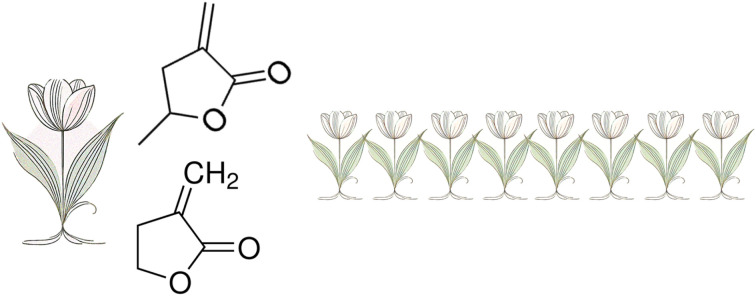

## Introduction

The transformation of the polymer industry into a more sustainable and renewable sector is a multifaceted challenge that hinges on several critical factors.^1^ Among these, the utilization of renewable feedstocks and the development of green polymerization routes stand out as key drivers. One approach involves mimicking the chemical structures of petroleum-derived monomers using renewable resources,^2^ allowing existing polymer synthesis procedures to remain largely intact. Alternatively, novel chemical structures derived from renewable sources can be designed to perform comparably, or even superiorly, to traditional petroleum-based polymer systems.^3^ A wide array of step-growth polymers, including polyamides,^4^ polyesters, polycarbonates,^5^ and epoxy resins,^6^ can now be synthesized using renewable feedstocks, achieving properties that meet or exceed those of their fossil-derived counterparts. Similarly, vinyl-functional renewable monomers offer promising opportunities to produce thermoplastics^7^ and thermosets.^8,9^ Successful adoption of renewable polymers in industry is shown by few industrial products. Beyond feedstock selection, the environmental impact of polymer synthesis can be significantly reduced through the adoption of renewable reaction pathways.^10^ This includes the use of green solvents,^11^ eco-friendly polymerization additives,^12^ and sustainable purification processes,^13^ all of which contribute to minimizing the ecological footprint of polymer manufacturing from monomer to final product.

Renewable monomers with lactone functionality have garnered significant attention due to their vast availability from sustainable feedstocks.^14^ Lactones, including lactide isomers, ε-caprolactone, β-butyrolactone, glycolide and valerolactone are widely recognized as prime candidates for ring-opening polymerization.^15^ This process yields polyesters with a broad spectrum of chemical and physical properties,^16,17^ making it a dominant route for renewable lactone-based monomers. Beyond ring-opening polymerization,^18^ lactone-containing monomers can also incorporate radical-polymerizable vinyl groups, enabling the synthesis of acrylic-like polymers with lactone-functional pendant units.^19^ For example, partially renewable polymer of mevalonic lactone methacrylate has emerged as a promising lactone bearing system which is depicted *via* radical photopolymerization.^20^ The mevalonic lactone moiety demonstrates exceptional reactivity toward nucleophiles for the design of highly functional polymers. Furthermore, coatings derived from this polymer exhibit enhanced reactivity, attributed to the inherent characteristics of the mevalonic lactone units. α-Methylene-γ-butyrolactone is present in tulip bulbs^21^ and has been studied in free radical^22^ controlled solution radical polymerization.^23^ Additionally, α-methylene-γ-butyrolactone has been subjected to controlled radical polymerization in green supercritical CO_2_ to afford biobased polymers with high sustainability metrics.^24^ Sebakhy and colleagues demonstrated enzymatic polymerization of α-methylene-γ-butyrolactone using surfactant-free latex method using horseradish peroxidase as initiator to achieve high conversion (98%) only in 3 hours.^25^ Films of α-methylene-γ-butyrolactone was prepared directly on diverse substrates *via* initiated chemical vapor deposition methodology, and controlled thickness and high conversion affords polymers with high transmittance and high glass transition temperature (164 °C) by this methodology.^26^ Alternatively, α-methylene-γ-valerolactone (MeGVL) has been an attractive monomer as it can be catalytically synthesized from biomass.^27^ It has been extensively explored for radical polymerization across various methods, including bulk and emulsion.^28,29^ MeGVL exhibits reactivity comparable to methacrylate-type monomers demonstrating its potential for adoption in the polymer industry. Additionally, depolymerization of vinyl lactone based polymer poly(γ-methyl-α-methylene-γ-butyrolactone) (PγMMBL) is evidenced to be highly selective and in higher yields compared to poly(methyl methacrylate) *via* thermolysis.^30^

**Scheme 1:**
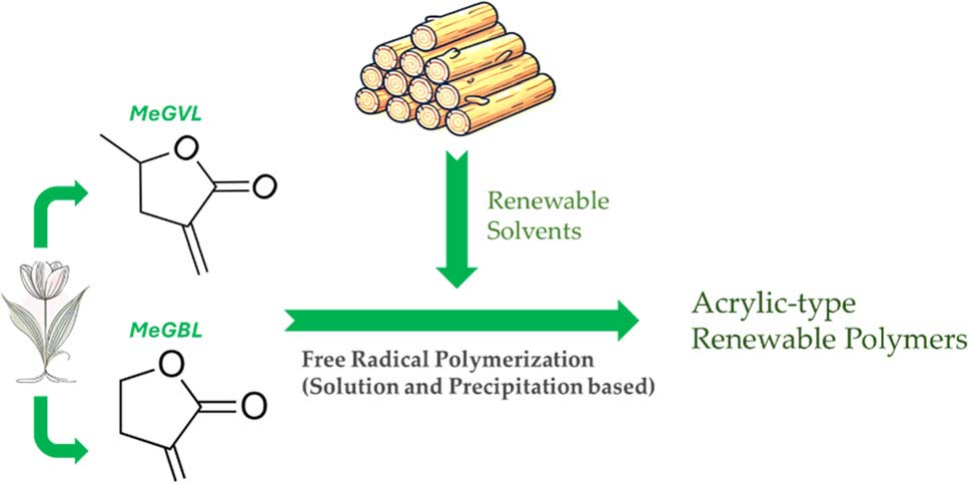
Depiction of synthetic methodology to manufacture acrylic-type renewable polymers *via* radical polymerization in renewable solvents.

In this study, we merge renewable vinyl lactone monomers with renewable solvents through radical polymerization experiments (Scheme 1). Emphasizing the use of potentially renewable components (excluding the radical initiator), potential efficiency of sustainable polymer manufacturing is examined. Both solution and precipitation polymerization pathways are accessible in presence of renewable solvents, which greatly influences the processing and cost of resulting polymers.

## Experimental details

### Materials

α-Methylene-γ-valerolactone (MeGVL, 97%, TCI Chemicals, CAS: 62873-16-9). α-Methylene-γ-butyrolactone (MeGBL, 95%, TCI Chemicals, CAS: 547-65-9), γ-butyrolactone (GBL, 99%, Merck, CAS: 96-48-0), γ-valerolactone (GVL, 99%, Merck, CAS: 108-29-2) azobisisobutyronitrile (AIBN, 98%, Merck, CAS: 78-67-1), 1-butanol (extra pure, Carl Roth, CAS: 71-36-3), 1,4-dioxane (anhydrous 99.8%, VWR, CAS: 123-91-1), 2-methyl tetrahydrofuran (MeTHF, 99%, Sigma-Aldrich, CAS: 96-47-9), basic aluminum oxide (Sigma-Aldrich, CAS: 1344-28-1), bioethanol (99%, Sydney Solvents, CAS: 64-17-5), Cyrene® (dihydrolevoglucosenone, 98.5%, Sigma-Aldrich, CAS: 53716-82-8), diethyl ether (anhydrous, Sigma-Aldrich, CAS: 60-29-7), dimethyl sulfoxide-d6 (Sigma-Aldrich, 99.9% contains TMS internal standard, CAS: 2206-27-1), isopropanol (IPA, 99%, Roth, 67-63-0). Nitrogen gas was supplied by Linde Gas from a cryogenic version stored in central facilities with a purity of 99%. Monomers MeGVL and MeGBL are subjected to basic aluminum oxide column treatment to remove inhibitor prior to polymerization reactions. Organic solvents are purified *via* vacuum supported rotavap distillation to remove any contaminant and inhibitor.

### Solution polymerization of MeGVL

2 g MeGVL is mixed with 10 mL solvent (GVL, GBL, MeTHF or Cyrene®) in presence of 10 mg AIBN in a 50 mL round bottom flask equipped with a magnetic stirrer. The solution is degassed using nitrogen gas for 15 minutes to eliminate any dissolved oxygen. Subsequently, solution is heated to 70 °C for 5 hours under continuous reflux. Upon completion of the reaction, the polymer is precipitated in 30 mL of bioethanol, filtered, and dried overnight in a vacuum oven at 70 °C. Resulting polymer is labelled as PMeGVL-s.

### Precipitation polymerization of MeGVL

2 g MeGVL is mixed with 20 mL alcohol (1-butanol, 2-phenylethanol or butanediol) in presence of 10 mg AIBN in a 50 mL round bottom flask equipped with a magnetic stirrer. The solution is degassed using nitrogen gas for 15 minutes to eliminate any dissolved oxygen. Subsequently, solution is heated to 70 °C for 5 hours under continuous reflux. During reaction, cloudy dispersion forms due to precipitation of so-formed polymer units. Upon completion of the reaction, mixture is filtered and dried overnight in a vacuum oven at 70 °C. Resulting polymer is labelled as PMeGVL.

### Precipitation polymerization of MeGBL

2 g MeGBL is mixed with 20 mL alcohol (1-butanol) in presence of 10 mg AIBN in a 50 mL round bottom flask equipped with a magnetic stirrer. The solution is degassed using nitrogen gas for 15 minutes to eliminate any dissolved oxygen. Subsequently, solution is heated to 70 °C for 5 hours under continuous reflux to increase operational safety. During reaction, cloudy dispersion forms due to precipitation of so-formed polymer units. Upon completion of the reaction, mixture is filtered and dried overnight in a vacuum oven at 70 °C. The resulting polymer is labelled as PMeGBL.

### Polymer film formation

10 wt% PMeGVL and PMeGBL solutions are prepared by dissolving a gram of polymer in 9 g of Cyrene. The solution is casted on a clean glass plate (microscope glass plate, width 25 mm length 75 mm) using razor blade approach to obtain a homogenous thickness (of around 100 μm). The glass plate is placed in a drying oven at 80 °C overnight for the removal of solvent. The resulting films are used for water contact angle and transmittance measurements.

### Characterization

Fourier transform infrared (FTIR) measurements are performed using a Spectrum 100 Optica FT-IR Spectrometer equipped with an attenuated total reflectance (ATR) accessory and a diamond crystal. The spectra are recorded in the range of 4000 to 550 cm^−1^ with a resolution of 1 cm^−1^ and 16 scans. Data processing is carried out with Origin software. The thermal stability of polymers are evaluated through thermogravimetric analysis (TGA) on a PerkinElmer TGA 4000 analyzer. All measurements are conducted under an inert nitrogen atmosphere (20 mL min^−1^). Samples ranging from 10 mg to 15 mg are heated from 25 °C to 700 °C at a rate of 10 °C min^−1^. Differential scanning calorimetry (DSC) is performed using a TA Instruments Model mDSC 250 system, with measurements taken in the range of 25–250 °C under nitrogen atmosphere, at a heating rate of 10 °C min^−1^. ^1^H-NMR measurements are collected on a 400 MHz Agilent NMR in d-DMSO solvent with 16 scans. Data was processed using MestReNova. SEC of polymer samples are collected in DMF using PSS 1260-Iso as a pump, a column system of PSS SDV column with a PSS SDV precolumn, PSS-SECcurity-VWD, and PSS-SECcurity-RID as detectors and a calibration with PMMA standards from PSS. Water contact angle measurements are conducted using the Attension Theta Optical Tensiometer by KSV Instruments using deionized (DI) water droplet carefully placed on sample surface. A digital image of the droplet is captured and analyzed using the Attension Theta software, which measured the left and right contact angles. The average contact angle is then calculated for each sample.

## Results and discussion

**Fig. 1:**
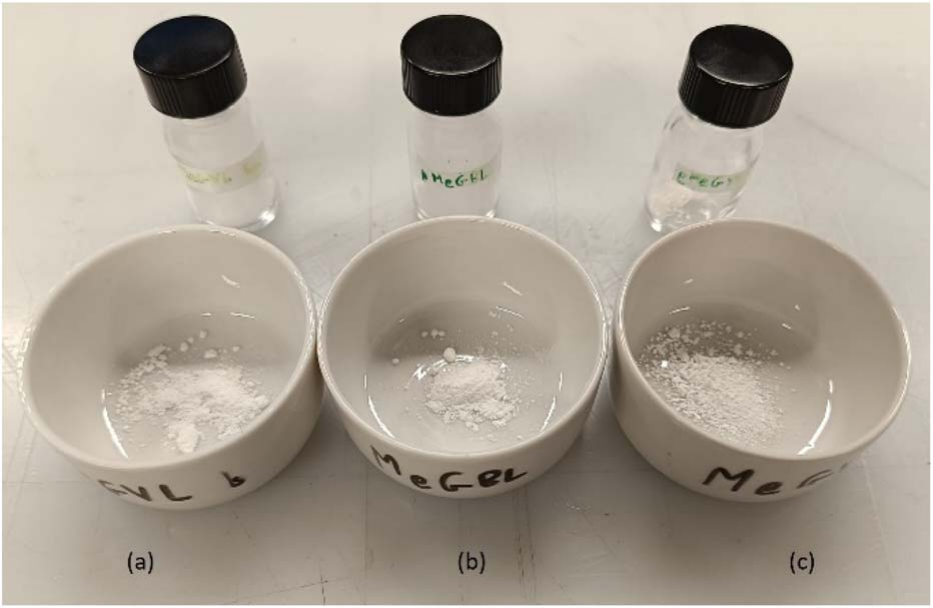
Image of synthesized renewable polymers (a) PMeGVL (b) PMeGBL (c) PMeGVL-s.

In this study, renewable vinyl lactone monomers, MeGVL and MeGBL, are polymerized *via* radical polymerization using organic renewable solvents as the reaction media. Depending on the polymer's solubility, either solution or precipitation polymerization routes are employed, as detailed in the experimental section. Precipitation polymerization offers a streamlined process,^31^ where the resulting polymers can be directly filtered and dried without additional purification steps.^32^ In this case, polymers synthesized *via* solution polymerization are precipitated in alcohol and dried only once to enable a direct comparison of yield and purity between the two methods. Notably, precipitation polymerization emerges as a highly scalable and sustainable strategy, as it minimizes the consumption of purification solvents^33^ and allows for efficient recycling of the reaction media.^34^ From an industrial perspective, this method presents a cost-effective approach, as it relies primarily on the initial investment in alcohol-based solvents, which can be reused multiple times hence reducing large-scale operational costs. The obtained polymers are shown in Fig. 1.

**Fig. 2:**
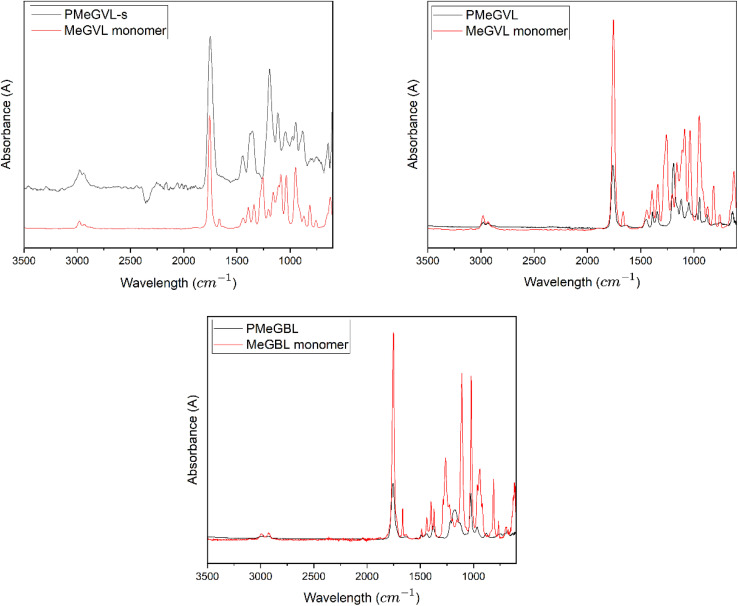
FTIR spectra of PMeGVL-s, pMeGVL and pMeGBL.

FT-IR spectroscopy is performed and confirms the polymerized structure of the GVL-based monomers. The spectra for PMeGVL-s, PMeGVL, and PMeGBL and their monomer counterparts are shown in Fig. 2. Absorption peaks at 2800–3000 cm^−1^ correspond to C–H stretching of the methylene (CH_2_) and methyl groups (CH_3_). The peaks in this region are more pronounced for PMeGBL and PMeGVL, suggesting a higher presence of alkyl groups compared to PMeGVL-s, where weaker peaks are observed. Around 1700 cm^−1^, C=O stretching is detected, and the peak at 1050 cm^−1^ highlights ether linkages, both supporting the presence of lactone rings. Additionally, the region near 1200 cm^−1^ corresponds to C–O stretching vibrations. Disappearance of C=C bond around 1600 cm^−1^ confirms successful polymerization and purification (removal of unreacted monomer) steps. Overall, the FT-IR spectra of the resulting polymers show no significant structural differences, confirming that the lactone functionality is retained after polymerization.

Technical use of polymers require high purities (ideally free of monomers and solvents), and commercial PMMA prepared by emulsion polymerization indicates high polymer purity as evidenced by ^1^H-NMR spectra (Fig. S1†).^1^H-NMR spectra of PMeGVL-s elucidates the formation of polymer structure in addition to impurities arising from the unreacted monomer and solvent, which fortifies the necessity to conduct additional purification step to reach certain technical quality (Fig. S2†). PMeGVL made *via* precipitation polymerization affords a rather clean spectrum with hydrogen signals arising from tertiary O–C_2_H (c), –CH_3_ (d) and –CH_2_ (a and b) are present (Fig. S3†). PMeGBL yields a similar spectrum with O–CH_2_ (c) and CH_2_ (a and b) in addition to pronounced O–CH_2_ peak arising from leftover butanediol (Fig. S4†). In short, precipitation pathway affords higher technical quality polymer compared to solution polymerization pathway, however longer drying step or higher drying temperatures could improve polymer purities.

Molecular weight analysis is performed in DMF using PMMA as standard, and obtained results are presented in Table 1. While polydispersities ranging from 1.4 to 1.7 indicates uncontrolled polymerization mechanism, the values are comparable to literature. Additionally, number average molecular weight obtained for PMeGVL-s is significantly lower than PMeGVL which affirms lower efficacy of Cyrene as polymerization medium. The role of solvents on the polymerization process of biobased lactones is shown by the works Sebakhy and colleagues,^23–26^ and despite being a renewable solvent Cyrene lowers polymerization efficiency of renewable lactones and generates purification difficulties in the final product enhancing the cost of polymer product. Molecular weight measurements of PMeGVL and PMeGBL demonstrates 31 000 and 27 000 (*M*_n_) g mol^−1^ with industrially acceptable conversion rates, however final molecular weights are lower compared to polymers made by emulsion polymerization with higher conversion values.^35^

**Table 1:** Molecular weight analysis of polymers

Polymer	Polymerization	Molecular weight (*M*_n_, g mol^−1^)	Polydispersity	Conversion (%)
PMeGVL-s	Solution	5500	1.7	72
PMeGVL	Precipitation	31 000	1.4	88
PMeGBL	Precipitation	27 000	1.5	83

**Fig. 3:**
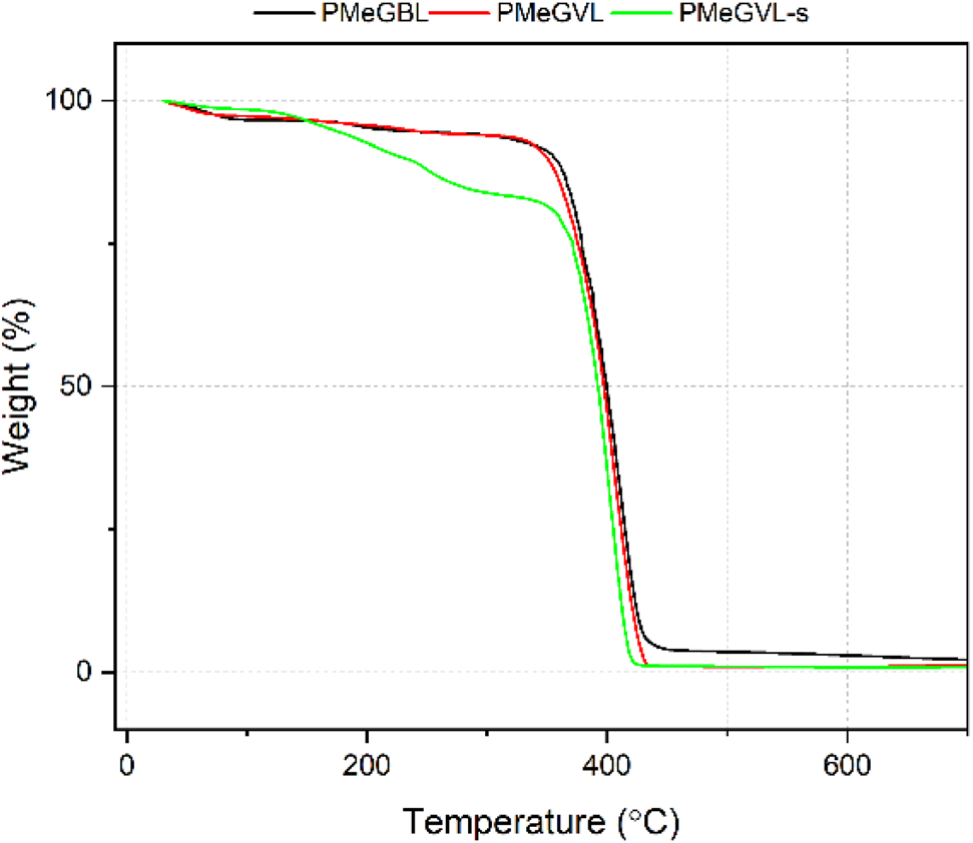
Thermogravimetric analysis results of polymer samples.

Thermal stability of polymers is studied by thermogravimetric analysis and can be seen in Fig. 3. Both PMeGBL and PMeGVL, represented by the black and red curve, possess similar thermal behavior with degradation starting around 250 °C, with almost complete degradation occurring by 600 °C. Most significant loss is observed around 380 °C, which corresponds to polymer degradation (Fig. S5†). Polymers elucidate negligible char yield evidenced at 600 °C. PMeGVL-s, represented by the green curve, starts a mass loss at a notably lower temperature around 140 °C. While the polymer degradation profile matches with PMeGVL, a significant mass loss at early stages can be attributed to impurities in polymer, especially unreacted monomer and solvent as well as oligomers. Thermal stability study confirms the efficacy of precipitation polymerization route to obtain higher purity polymers for further processing steps.

**Fig. 4:**
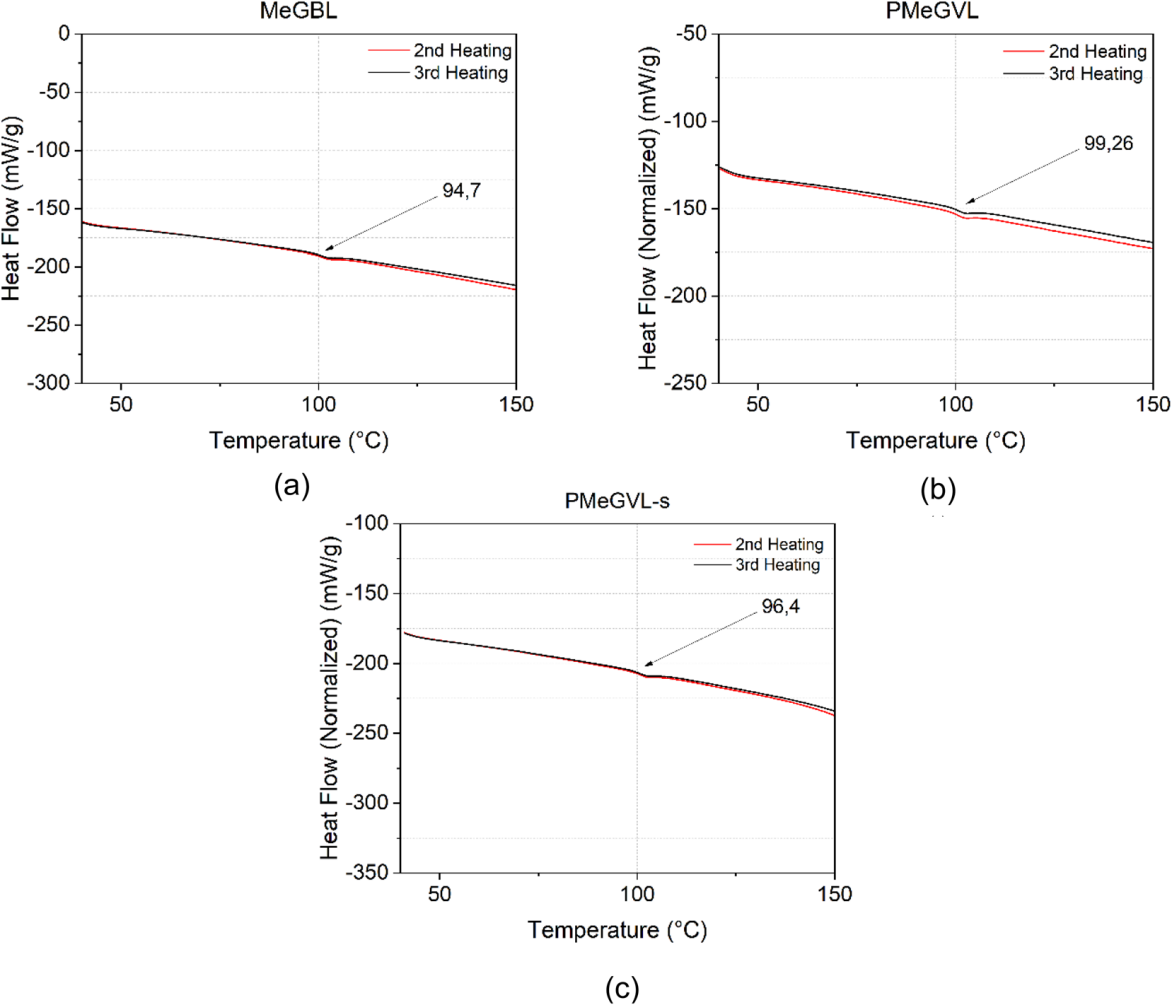
2nd and 3rd DSC runs of (a) PMeGBL, (b) PMeGVL and (c) PMeGVL-s.

DSC analysis is used to determine the glass transition temperature (*via* onset) of the synthesised amorphous thermoplastic polymers. For PMeGBL, a glass transition temperature is observed at 94.7 °C (Fig. 4a). The PMeGVL graph exhibits a similar pattern, with a glass transition temperature of 99.26 °C (Fig. 4b). For PMeGVL-s, a glass transition is noted 96.4 °C (Fig. 4c). Overall, the second and third heating scans for all three materials show minimal variation, suggesting thermal and structural stability of lactone bearing renewable acrylic polymers. Additionally, *T*_g_ values are close to the ones of PMMA,^36^which is 103 °C (Fig. S6†) indicating a potential for substitution of PMMA in industrial applications for so-formed renewable polymers.

A selection of solvents is studied to assess the solubility of so-formed polymers *via* precipitation route, as given in Table 2. Since solution polymerization route affords polymers with lower purity compared to precipitation route, following tests will focus on using purer precipitation route. Both polymers demonstrate high solubility in Cyrene, DMF and GVL whereas THF resulted in poor solubility contrary to literature. Based on these results, polymer films are manufactured as detailed in experimental section.

**Table 2:** Solubility chart of resulting polymers

Solvent	PMeGVL	PMeGBL
THF	∼^a^	∼^a^
MeTHF	∼^a^	∼^a^
Cyrene	+	+
1,4 Dioxane	−	−
Chloroform	∼^b^	∼^b^
DMF	+	+
GVL	+	+
Water	−	−
Diethyl ether	−	−
Toluene	−	−

a Soluble at temperatures above 40 °C at concentrations of 2 wt%.

b Soluble at temperatures above 30 °C at concentrations of 2 wt%.

Polymer films are manufactured and used for water contact angle and transmittance measurements. The water contact angle (WCA) measurements (Table 3 and Fig. S7†) provide insights into the surface wettability. The average WCA for PMeGVL is 62.36° and 63.15°, indicating a moderately hydrophilic surface with small standard deviations (2.27 and 2.53), suggesting consistent surface properties. In contrast, PMeGBL shows lower average contact angles of 51.91° and 50.88°, highlighting a more hydrophilic surface with slightly larger standard deviations (3.09 and 2.39), implying minor surface heterogeneity. When compared to PMMA, which typically has a WCA of approximately 70°,^37^ both PMeGVL and PMeGBL exhibit greater hydrophilicity due to lactone pendant groups. While *T*_g_ values are lower compared to renewable PMeGBL films made by emulsion,^25^ our method enables film extrusion technique for continuous polymer film manufacturing.

**Table 3:** Water contact angle results of PMeGVL and PMeGBL surfaces

	PMeGVL		PMeGBL	
	*θ*° (left)	*θ*° (right)	*θ*° (left)	*θ*° (right)
	64.66	64.95	49.02	47.06
	62.45	63.50	56.30	54.39
	58.04	58.26	47.91	50.48
	63.49	65.18	53.95	52.04
	63.16	63.85	52.37	50.46
Average	62.36	63.15	51.91	50.88
*σ*	2.27	2.53	3.09	2.39

**Fig. 5:**
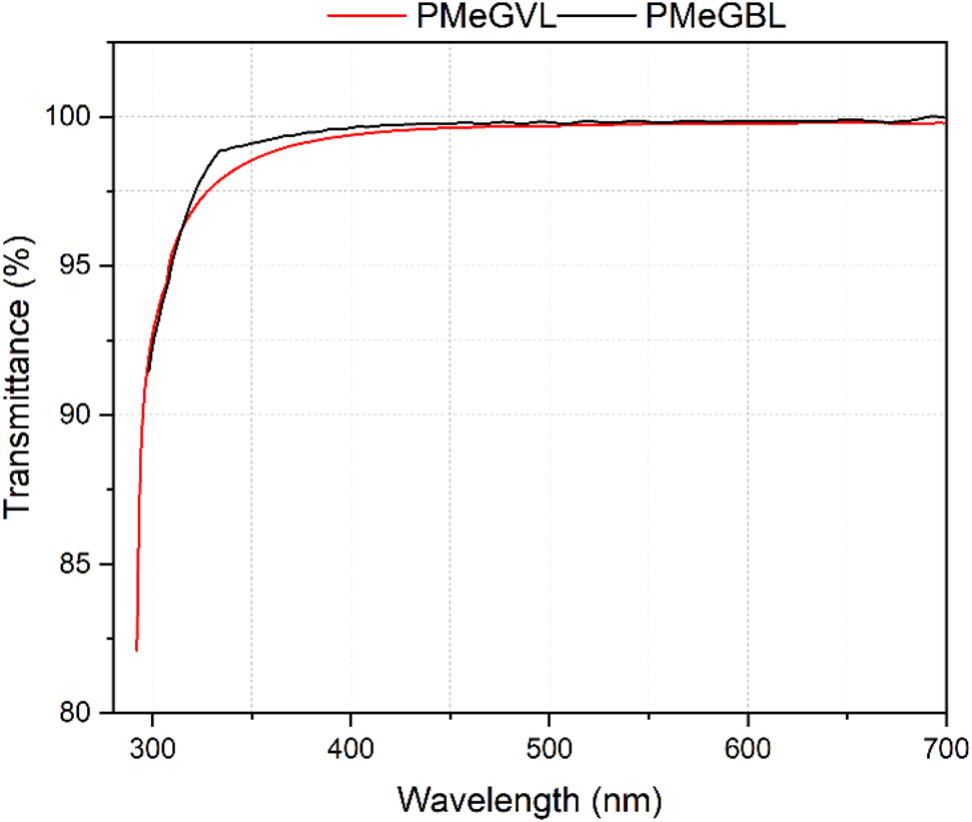
Transmittance measurement of casted polymer films within 300–700 nm range.

Amorphous polymers in high purity are known for their transparency due to lack of light-scattering crystalline domains, and they address applications where transparent look is important. Renewable PMeGVL and PMeGBL films possess high transparency as evidenced by transmittance measurements (Fig. 5 and S8†). While films show low transmittance within 300–350 nm range, in the visible range of 450–700 nm films exhibit up to 98% transparency.

## Conclusion

The transition to a fossil-free polymer industry remains crucial for enhancing the valorization of biomass resources and fostering sustainable material solutions. MeGVL and MeGBL, which are abundantly available in tulip bulbs or can be synthesized from biomass *via* efficient two-step catalytic reactions, serve as key building blocks for this transition. Coupled with the growing commercial availability of certified renewable solvents derived from biomass, these monomers enable the synthesis of novel acrylic-like lactone-functional polymers with a glass transition temperature of approximately 99 °C. These polymers exhibit thermal stability and optical transparency comparable to PMMA, making them promising candidates for medium-performance polymer applications. Additionally, precipitation polymerization in alcohols yields high-quality polymer products while minimizing purification steps, and the reaction media can be efficiently reused, further enhancing process sustainability. By maximizing the use of biomass-derived synthons, this project sets a base for truly green polymer production. Future studies can explore additional functionalities, such as controlled biodegradation and polymer post-modifications *via* lactone chains to expand the scope of these renewable polymers.

## Conflicts of interest

Authors has a Dutch patent (no: 2032709) on these finding entitled “Green production of sustainable polylactones”.

## Supplementary Material

RA-015-D5RA02151K-s001

## Data Availability

Data generated in this work is currently confidential due to a pending patent application.
