# Supplementary material for: Solution and precipitation based radical polymerization of renewable vinyl lactones in renewable solvents
Source: RSC Adv. 2025 Jun 25;15(27):21659–65. doi: 10.1039/d5ra02151k (PMC12188522; doi:10.1039/d5ra02151k)
Supplement: RA-015-D5RA02151K-s001 [file RA-015-D5RA02151K-s001.pdf]

Supplementary Information (SI) for RSC Advances.
This journal is © The Royal Society of Chemistry 2025

Supporting Information For

**Solution and Precipitation Based Radical Polymerization of Renewable Vinyl Lactones in Renewable Solvents**

Dimitrios Apostolidis, William E. Dyer, Clemens A. Dransfeld, Baris Kumru*

Aerospace Structures & Materials Department, Faculty of Aerospace Engineering, Delft University of Technology, 2629 HS Delft, Netherlands

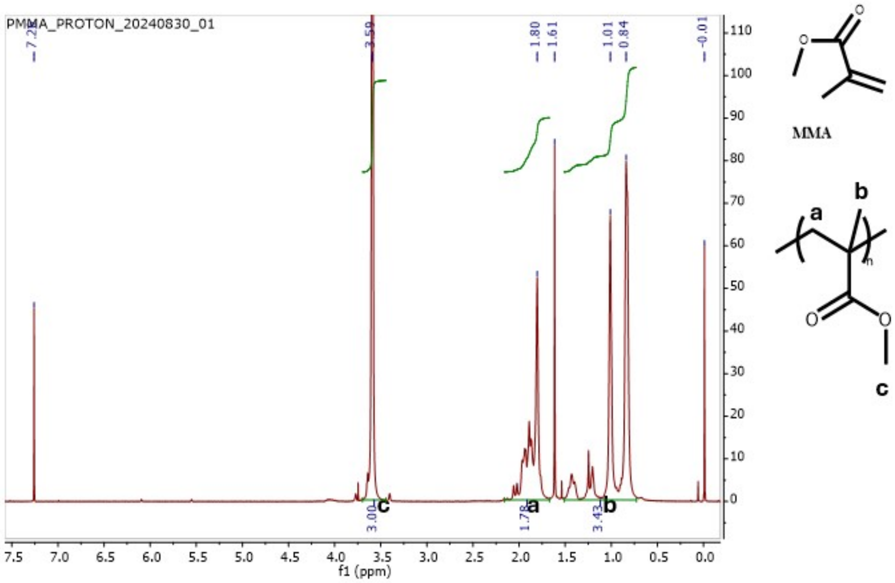


*Figure S1. 1H-NMR of commercial PMMA.*

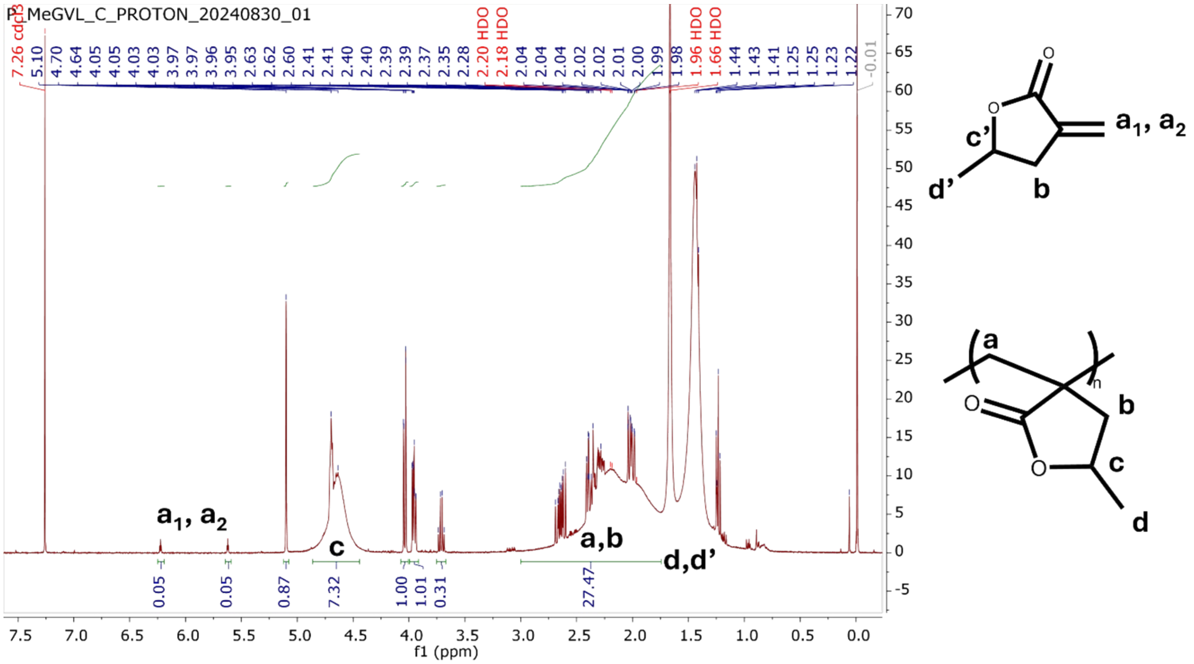


Figure S2. 1H-NMR of PMeGVL-s.

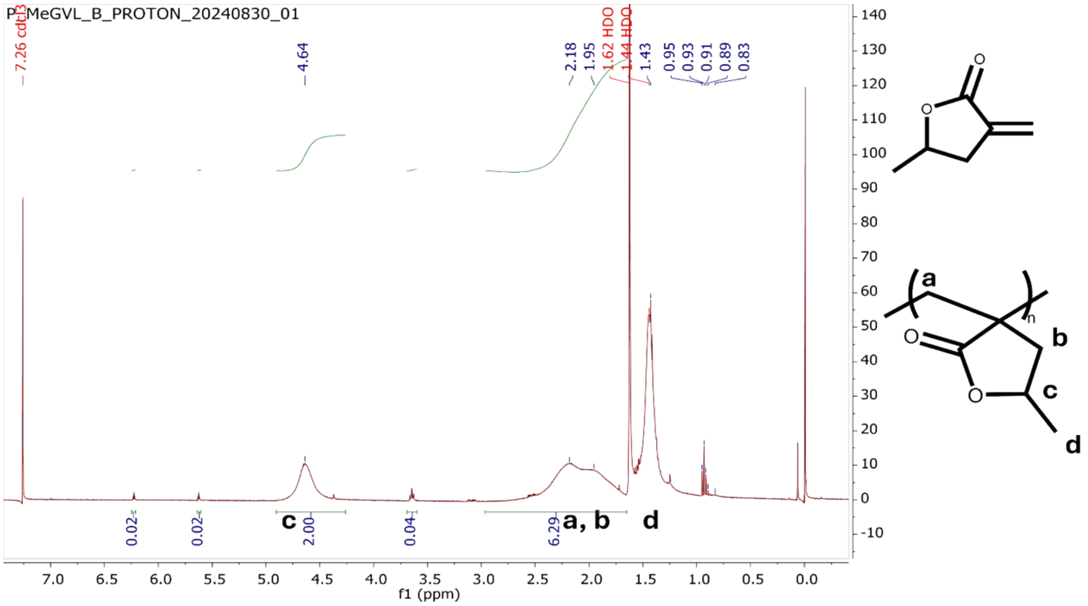


*Figure S3. 1H-NMR of PMeGVL.*

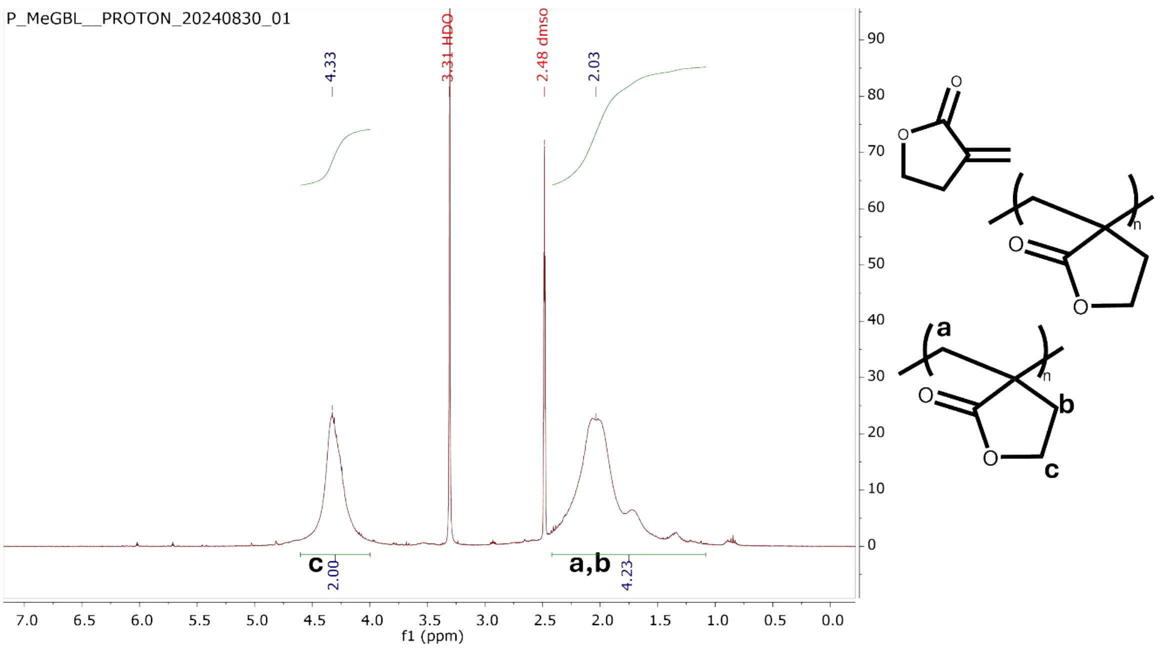


*Figure S4. 1H-NMR of PMeGBL.*

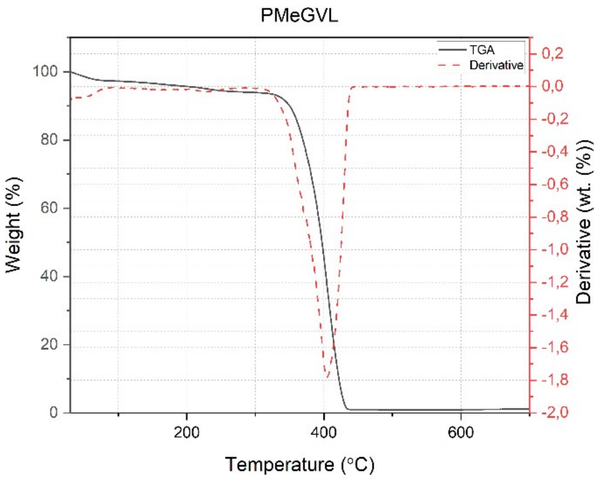


(a)

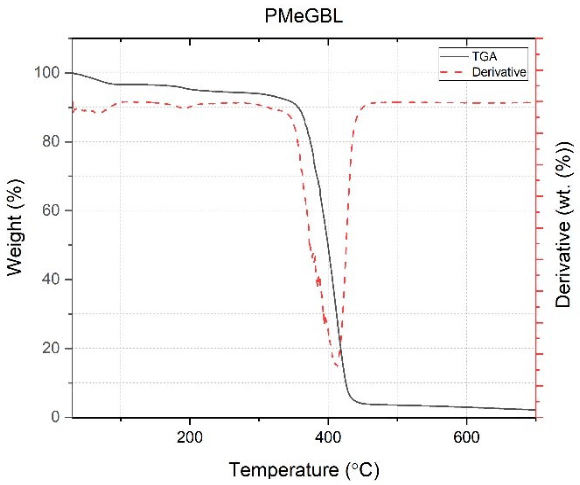


(b)

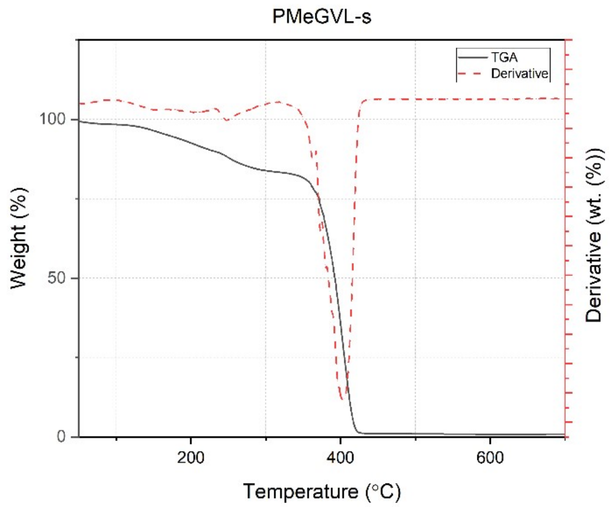


(c)

Figure S5. TGA and DTG graphs of (a) PMEGVL, (b) PMeGBL and (c) PMeGVL-s.

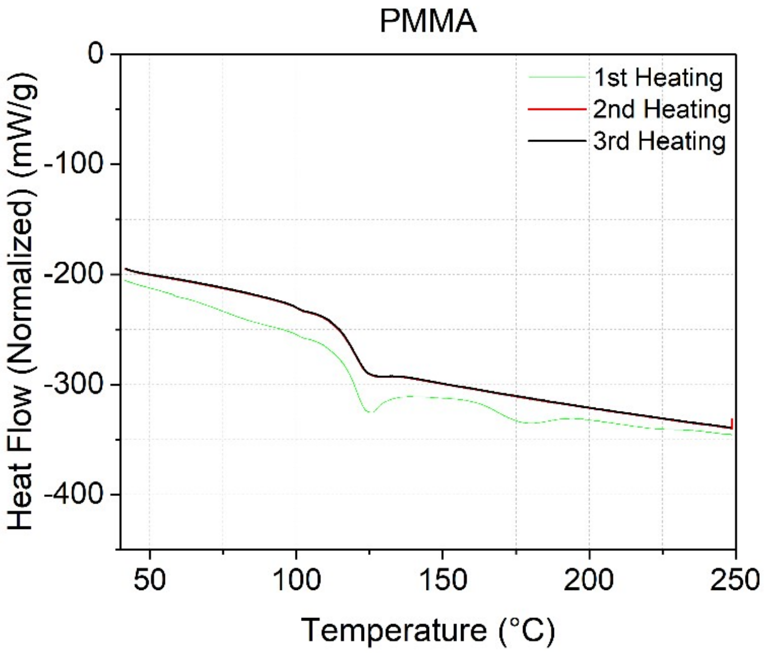


*Figure S6. DSC diagram of commercial PMMA.*

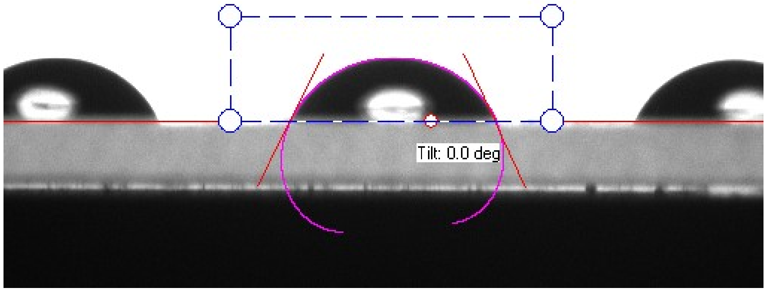


*Figure S7. Digital image of an exemplary WCA on casted PMeGVL film.*

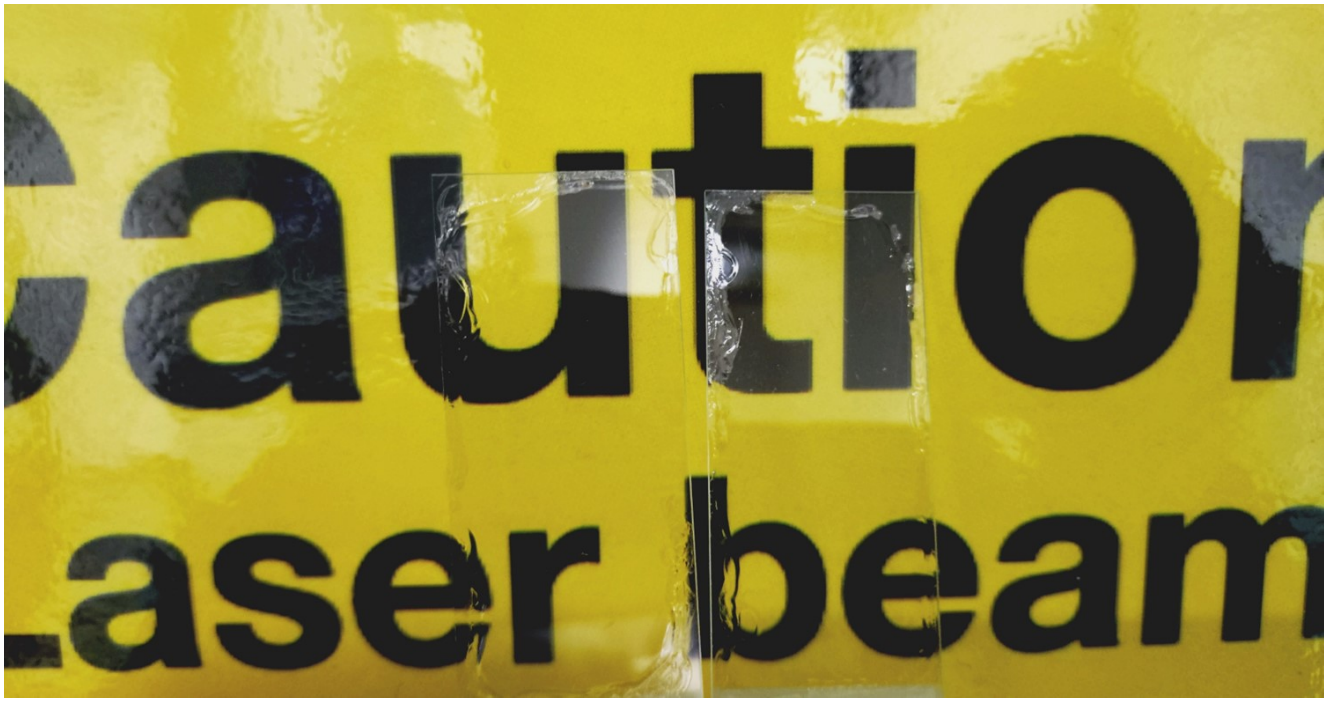

Figure S8. Digital image of casted polymer films (left: PMeGVL, right: PMeGBL).
